# Human Bacterial Artificial Chromosome (BAC) Transgenesis Fully Rescues Noradrenergic Function in Dopamine β-Hydroxylase Knockout Mice

**DOI:** 10.1371/journal.pone.0154864

**Published:** 2016-05-05

**Authors:** Joseph F. Cubells, Jason P. Schroeder, Elizabeth S. Barrie, Daniel F. Manvich, Wolfgang Sadee, Tiina Berg, Kristina Mercer, Taylor A. Stowe, L. Cameron Liles, Katherine E. Squires, Andrew Mezher, Patrick Curtin, Dannie L. Perdomo, Patricia Szot, David Weinshenker

**Affiliations:** 1 Department of Human Genetics, Emory University School of Medicine, Atlanta, Georgia, United States of America; 2 Emory Autism Center, Department of Psychiatry and Behavioral Sciences, Emory University School of Medicine, Atlanta, Georgia, United States of America; 3 Center for Pharmacogenomics, College of Medicine, The Ohio State University, Columbus, Ohio, United States of America; 4 Graduate Program in Genetics and Molecular Biology, Emory University, Atlanta, Georgia, United States of America; 5 MIRECC, VA Puget Sound Health Care System, Seattle, Washington, United States of America; 6 Department of Psychiatry and Behavioral Sciences, University of Washington, Seattle, Washington, United States of America; Hudson Institute, AUSTRALIA

## Abstract

Dopamine β-hydroxylase (DBH) converts dopamine (DA) to norepinephrine (NE) in noradrenergic/adrenergic cells. DBH deficiency prevents NE production and causes sympathetic failure, hypotension and ptosis in humans and mice; DBH knockout (*Dbh -/-*) mice reveal other NE deficiency phenotypes including embryonic lethality, delayed growth, and behavioral defects. Furthermore, a single nucleotide polymorphism (SNP) in the human *DBH* gene promoter (-970C>T; rs1611115) is associated with variation in serum DBH activity and with several neurological- and neuropsychiatric-related disorders, although its impact on DBH expression is controversial. Phenotypes associated with DBH deficiency are typically treated with L-3,4-dihydroxyphenylserine (DOPS), which can be converted to NE by aromatic acid decarboxylase (AADC) in the absence of DBH. In this study, we generated transgenic mice carrying a human bacterial artificial chromosome (BAC) encompassing the *DBH* coding locus as well as ~45 kb of upstream and ~107 kb of downstream sequence to address two issues. First, we characterized the neuroanatomical, neurochemical, physiological, and behavioral transgenic rescue of DBH deficiency by crossing the BAC onto a *Dbh -/-* background. Second, we compared human *DBH* mRNA abundance between transgenic lines carrying either a “C” or a “T” at position -970. The BAC transgene drove human *DBH* mRNA expression in a pattern indistinguishable from the endogenous gene, restored normal catecholamine levels to the peripheral organs and brain of *Dbh -/-* mice, and fully rescued embryonic lethality, delayed growth, ptosis, reduced exploratory activity, and seizure susceptibility. In some cases, transgenic rescue was superior to DOPS. However, allelic variation at the rs1611115 SNP had no impact on mRNA levels in any tissue. These results indicate that the human BAC contains all of the genetic information required for tissue-specific, functional expression of *DBH* and can rescue all measured *Dbh* deficiency phenotypes, but did not reveal an impact of the rs11115 variant on *DBH* expression in mice.

## Introduction

Successful gene therapy, in which introduction of an external DNA construct replaces an absent or malfunctioning gene, will depend in large part on ensuring specific targeting of gene expression to appropriate cell types. The rare human syndrome of dopamine β-hydroxylase (DBH) deficiency results in severe orthostatic hypotension, ptosis, and high levels of circulating dopamine (DA), which reflect the inability of noradrenergic cells to synthesize norepinephrine (NE), resulting in absence of sympathetic noradrenergic tone [1, 2]. Human DBH deficiency results from rare deleterious mutations in the *DBH* gene, which lead to absent or inadequate expression of DBH protein [3].

Targeted disruption of *Dbh* in mice produces a precise model of DBH deficiency [4]. The observations that *Dbh -/-* mice are born in substantially smaller proportions than predicted by Mendelian expectations, and that surviving pups exhibit almost 100% mortality within the first week of life [4], highlight the essential roles of DBH and NE in development and survival. Prenatal and perinatal administration of L-3,4-dihydroxyphenylserine (DOPS), a hydroxylated precursor that is converted to NE by the enzyme aromatic acid decarboxylase (AADC), restores NE synthesis and rescues survival of *Dbh -/-* animals. The pre-natal mortality associated with the *Dbh-/-* phenotype arises from cardiovascular instability, which for unclear reasons stabilizes shortly after birth, thus allowing withdrawal of DOPS support. Once DOPS-treated *Dbh -/-* mice are born, they survive without pharmacological intervention, thereby allowing study of this interesting mutant in adulthood in the absence of NE. *Dbh -/-* mice have been a useful tool in a variety of investigations of the role of NE in behavior, including neurologically and psychiatrically relevant phenotypes such as arousal [5–7], seizure susceptibility [8], anxiety- and depression-like behaviors [9, 10], learning and memory [11, 12] and a variety of responses to drugs of abuse [13–18].

DBH activity can be measured in human serum, where the wide variation in enzyme activity observed in the population reflects variations in levels of DBH protein derived from sympathetic noradrenergic neurons and neurosecretory cells of the adrenal medulla [19]. Serum DBH level is a genetic trait largely refractory to environmental influences [19–21]. Genotype at -970C>T (rs1611115), a single nucleotide polymorphism (SNP) residing 970 bp upstream of the transcriptional start site of the *DBH* gene, accounts for 30–50% of the variance in serum DBH levels [22]. The C allele associates with substantially higher serum DBH activity than the T allele, an observation that has been repeatedly replicated in human samples of diverse ancestry [22–25]. However, because this SNP lies in the vast presumptive promoter region that contains many other variants, demonstrating a cause-and-effect relationship has been difficult. A genome-wide association study (GWAS) of serum DBH levels recently demonstrated that -970 C>T associates with variation in serum DBH more strongly than any other marker tested across the genome [26]. The foregoing observations prompted the hypothesis that -970C>T associates with variation in serum DBH activity because it alters expression of the *DBH* gene, which should be detectable at the mRNA level. Chen and colleagues [27] tested the function of -970C>T in transient transfection assays of reporter plasmids containing each allele in the context of approximately 3 kb of *DBH* upstream sequence. Their results supported the hypothesis that -970C>T alters gene expression, but interestingly, yielded data suggesting the T allele associates with higher reporter expression than the C allele, a result that is opposite to that expected from association studies of human serum DBH.

Barrie and colleagues [28] examined DBH mRNA expression in human tissues, providing evidence that -970C>T associates with variation in DBH mRNA expression in liver (where DBH is presumed to be present by virtue of hepatic sympathetic innervation), with the T allele associating with lower DBH mRNA expression. However, there was no evidence for an association of -970C>T with variation in DBH mRNA expression in the brain or adrenals. Thus, available evidence suggests that the influence of -970C>T on expression of *DBH* may be specific to sympathetic neurons. Interestingly, the association of variation at -970C>T with *DBH* expression at the mRNA level in transient transfection assays [25] is in the opposite direction expected from its association with serum DBH activity and in human liver [28]. The reason for this set of observations remains unknown, although it is likely that the transient transfection constructs lacked all of the pertinent regulatory sequences present *in vivo*.

The present study examined the *in vivo* function of -970C>T within the context of extensive human genomic sequence naturally surrounding the variant. We used bacterial artificial chromosome (BAC) constructs differing in sequence only at -970C>T to restore expression of DBH in *Dbh -/-* mice, to test three hypotheses. First, that introduction of BAC constructs containing the full human *DBH* gene and extensive surrounding sequence would rescue DBH expression in *Dbh -/-* mice, resulting in correction of abnormal phenotypes associated with absence of DBH and NE. Second, that the constructs would drive anatomically appropriate expression of DBH. Third, that BAC constructs containing the C allele at *DBH* position -970 would drive greater expression of DBH within noradrenergic neurons than those containing the T allele. Our results confirm the first two hypotheses, but not the third.

## Materials and Methods

### Animals

*Dbh -/-* mice on a mixed C57BL6/J and 129SvEv background were descendants of those produced by Thomas et al. [4] and maintained on a standard 12 hour light/dark cycle with food and water available ad libitum except during behavioral testing. All procedures were conducted in strict accordance with the NIH Guide for the Care and Use of Laboratory Animals and approved by the Emory University Institutional Animal Care and Use Committee.

### Bacterial artificial chromosome cloning

The human *DBH* gene, on chromosome 9q34.2, has 12 exons and is ~23 kb in length from transcriptional start to transcriptional end. We chose the commercially available RP11-746P3 BAC (~ 175 kb) because it is likely to contain sufficient sequence both upstream (~ 45 kb) and downstream (~ 107 kb) from *DBH* to include all necessary elements required for correct expression. We obtained the BAC clone from BACPAC Resources, Children’s Hospital Oakland (Oakland, CA), isolated and purified the BAC using the Qiagen Large-Construct kit, and sequenced all exons and intron-exon boundaries for DBH to ensure it retained the full-length gene. We also sequenced the upstream sequence containing position –970 and determined that the original BAC contained a “T” at position –970. We successfully inserted a unique AsiI restriction enzyme site into the BAC vector backbone for later linearization. We then created a second, “C” form of the BAC, by converting the “T” to a “C” at position –970 of the *DBH* gene, using the protocol described by Yang and Shara [29]. Briefly, the BAC was stably transfected into a strain of bacteria carrying recombinatory genes under the control of a temperature-sensitive promoter. We then transformed the cells with our custom-designed oligonucleotide carrying the “C” allele and adjusted the temperature to allow recombination to occur. PCR served to screen for positive clones. Sequencing confirmed that the “C” and “T” BAC transgene constructs were complete and ready for pronuclear injection.

### Preparation of BAC transgenic mice

BACs were purified with the Qiagen Large-Construct kit, linearized with AsiI, and isolated on a pulse-field gel. The BAC DNA was then used to generate BAC transgenic mice on a FVB background in the Emory University Mouse Transgenic and Gene Targeting Core Facility (http://www.cores.emory.edu/tmc/index.html) using standard transgenic methods. PCR on tail-snip DNA using human-specific primers corresponding to multiple sites on the transgene confirmed that the human insert within the BAC had fully integrated, and that all exons of human *DBH* were intact. We used 4 transgenic founders (two with the “C” allele and two with the “T” allele) to establish 4 independent transgenic lines for further characterization. We then crossed each of these lines onto a *Dbh -/-* background, which will be referred to as *Dbh -/- BT* lines. We chose one of the “T” lines for extensive behavioral, physiological, and neurochemical analysis. The other “T” line and the 2 other “C” lines were used exclusively to determine the consequences of the -970C>T (rs1611115) polymorphism on mRNA expression.

### Survival of *Dbh -/- BT* mice

*Dbh -/- BT* males were crossed to *Dbh +/-* females. Offspring were genotyped at weaning (21 days), and the fraction of mice of each genotype was compared to the expected Mendelian ratio. For comparison between the efficacy of genetic and pharmacological rescue of the *Dbh -/-* lethal phenotype, we also crossed *Dbh -/-* without the BAC transgene with *Dbh +/-* females, as described [30]. Briefly, pregnant *Dbh +/-* females were given the adrenergic receptor agonists isoproterenol and phenylephrine (20 μg/ml each) + vitamin C (2 mg/ml) from E9.5-E14.5, and DOPS (2 mg/ml + vitamin C 2 mg/ml) from E14.5-birth in their drinking water.

### Ptosis

Ptosis was determined in adult (3–6 months) mice at a fixed distance and measuring the maximum separation between the eyelids, as described [30].

### Novelty-induced locomotor activity

Mice were placed in locomotion recording chambers (transparent Plexiglas cages placed into a rack with 7 infrared photobeams spaced 5 cm apart; San Diego Instruments Inc., La Jolla, CA), and ambulations (consecutive beam breaks) were recorded for 30 min.

### Flurothyl-induced seizures

Mice were placed in an air-tight Plexiglas chamber, and the volatile convulsant Bis(2,2,2-trifluoroethyl) ether (flurothyl; Sigma Aldrich, St. Louis, MO) was dripped (20 μl/min) onto filter paper from which it vaporized. Latency to generalized (tonic-clonic) seizure was measured, as described [8].

### Catecholamine measurement by HPLC

Levels of NE and DA were quantified using high-performance liquid chromatography coupled with electrochemical detection using procedures similar to those described previously [31]. Briefly, mice were euthanized by CO2 asphyxiation, and brain, adrenal, and heart were isolated and frozen on dry ice. Frozen tissue samples were initially prepared by adding 200 μl of ice-cold 0.1 N perchloric acid containing 0.04% sodium metabisulfite and then centrifuged at 13.2 x 1000 r.p.m. for 10 min at 4°C. A 50 μl aliquot of each sample was placed into a microcentrifuge tube and loaded into a refrigerated autosampler (G1329A, Agilent Technologies, Santa Clara, CA), which injected 15 μl of each sample onto an Ultrasphere ODS 250 x 4.6 mm column, 5 μm (Beckman Coulter, Fullerton, CA) at a constant flow rate of 1.0 ml/min using a mobile phase consisting of 0.1 mM ethylenediaminetetraacetic acid, 0.8 mM sodium octyl sulfate, 0.7% phosphoric acid, and 5% acetonitrile (pH 2.6). Separated analytes were detected and quantified using a Coulochem III detector (ESA Inc., Chelmsford, MA), a high sensitivity analytical cell (channel 1, -150 mV; channel 2, +300 mV; model 5011A, ESA Inc.), and a guard cell (+400 mV; model 5020, ESA Inc.). A set of standards containing experimenter-prepared concentrations of NE and DA (50–1000 nM) were analyzed in duplicate along with experimental samples. ChemStation chromatography software (Agilent Technologies) generated chromatograms for each sample analyzed and calculated area under the curve for each peak. Standards were used to generate a standard plot (area under the curve X analyte concentration) from which the estimated concentration in experimental samples was extrapolated.

### *In situ* hybridization

To assess anatomic specificity of *DBH* expression, we employed *in situ* hybridization. C57Bl6/J wild-type (WT) (Jackson Laboratories, Bar Harbor, ME) and *Dbh -/- BT* mice were euthanized by CO2 asphyxiation, and brains were removed and frozen on dry ice. Sixteen-micrometer coronal sections containing the substantia nigra/ventral tegmental area (SN/VTA) and locus coeruleus (LC) were cut on a cryostat and mounted onto Fisher Superfrost slides (Fisher Scientific, Houston, TX). Slides were stored at -80°C until assayed. Tissue preparation and labeling of the mouse TH and human DBH oligonucleotides was performed as described previously [32]. The mouse TH oligonucleotide probe was a 48 base probe complementary to nucleotides 1351–1398 of the TH mRNA [33]. The human DBH oligonucleotide is composed of two different 51-base oligonucleotides (regions 478–529 and 1339–1390) of the human DBH sequence [34]. Each oligonucleotide was 3’-end-labeled with [33P]dATP (New England Nuclear, Boston, MA) using terminal deoxyribonucleotidyl transferase (Invitrogen, Piscataway, NJ) and then purified with Illustra MicroSpin G-25 Columns (GE Healthcare, Piscataway, NJ). The mouse TH hybridization buffer contained 0.35 X 10^6^ cpm/50 μl, and the human DBH hybridization buffer contained 1.32 X 10^6^ cpm/50 μλ. Slides were washed as described in detail in previously published work for the oligonucleotide probes [32, 35] and apposed to film (Eastman Kodak, Rochester, NY) at room temperature for 18 hours for mouse TH and 4 days for human DBH.

### Reverse-transcription PCR

Mice were euthanized by CO2 asphyxiation, and brain (LC microdissected), adrenal, heart, liver, and lung were isolated and frozen on dry ice. Tissue was homogenized in TRIzol, and RNA was extracted as previously described including column purification and DNase treatment to remove residual gDNA [36]. Due to the small amount of tissue available for the adrenal samples, 1 μg of glycogen was added to aid in RNA recovery. We measured the RNA integrity via Bioanalyzer (Agilent Technologies) for a subset of samples for each tissue type and genotype group. Concentrations were measured using the RNA or DNA quantitation reagent on the Qubit Fluorometer (Life Technologies) and diluted to equal concentrations. cDNA was synthesized via reverse transcription with SuperScript III (Invitrogen), oligo-dT, and gene-specific primers for human DBH and SARDH.

### Quantitative real-time PCR (qRT-PCR)

mRNA expression was measured by qRT-PCR with a 7500 Fast Real-Time PCR System (Life Technologies) with the following primers: DBH_F: 5′GACGCCTGGAGTGACCAGAA, DBH_R_RNA: 5′CAGTGACCGGAACGGCTC. Reactions were prepared in duplicate in 10 ul volumes with Fast SYBR Green Master Mix (Applied Biosystems). After PCR, amplification plots were inspected, threshold values were set to 0.2 using the 7500 Software v2.0.5 (Applied Biosystems) and threshold cycle numbers (Ct) were obtained. We also designed multiple 150–190 bp product primer sets along the BAC to test for the presence of copy number variation. The mean Ct for the four sets was used to normalize the RNA expression values for each mouse. The primers were BAC2F: 5′GCCTGCCCTCTGCCAAC, BAC2R: 5′CCTGGGTGGGACTTGGAAC, BAC9F: 5′TGTCCACTTGCAGCACAGC, BAC9R: 5′AGGAGCTTGGAAAACCGGA, BAC31F: 5′TCACACCATGCTGCCACC, BAC31R: 5′CGACTTTGCTCTTGCCTGC, BAC37F: 5′GCTGTTTACCCCACGCCA and BAC37R: 5′GGAATGATGCTGGGTGGTG. For these experiments, DNA template was extracted from brain via overnight digestion, ethanol precipitation, and phenol chloroform extraction [37].

## Results

### The human RP11-746P3 BAC contains regulatory elements sufficient to drive anatomically accurate human DBH mRNA expression in the mouse

**Fig 1:**
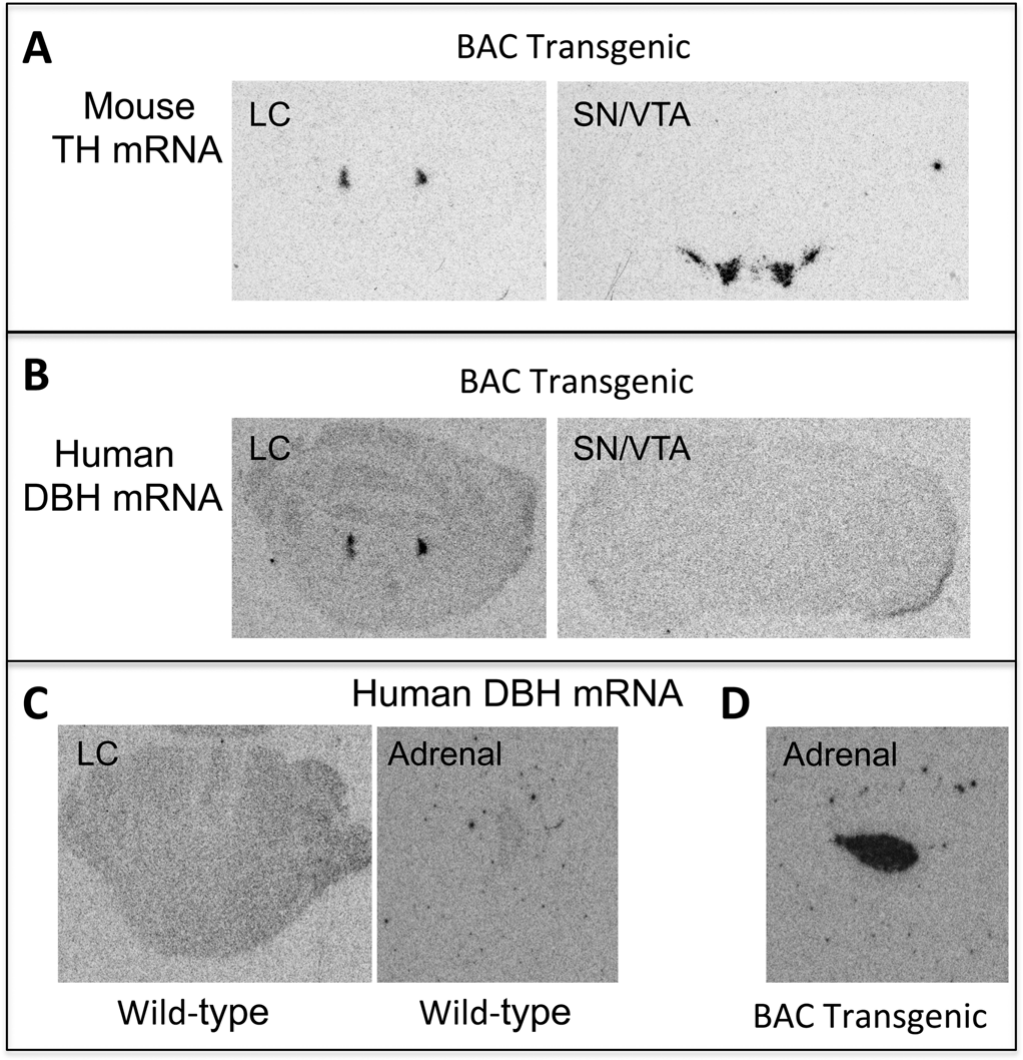
A human BAC transgene drives specific Dbh expression to the locus coeruleus and adrenal gland. Shown are representative examples of human dopamine β-hydroxylase (DBH) and mouse tyrosine hydroxylase (TH) mRNA expression in adrenal gland and brain sections from C57Bl6/J wild-type and BAC transgenic mice containing the noradrenergic locus coeruleus (LC, corresponding to Figure 75 in the Mouse Brain Atlas) and the dopaminergic substantia nigra pars compacta/ventral tegmental area (SN/VTA, corresponding to Figure 55 in the Mouse Brain Atlas[38]).

*DBH* mRNA is normally expressed exclusively in noradrenergic/adrenergic cells, while tyrosine hydroxylase (TH) mRNA is expressed in all catecholaminergic neurons. Fig 1 shows in situ hybridization micrographs from wild-type C57Bl6/J and *Dbh -/- BT* mice. As expected, mouse TH is expressed in both midbrain DA neurons and LC neurons of *Dbh -/-* BT mice. By contrast, human DBH mRNA is restricted to LC neurons in the brain and the adrenal medulla of *Dbh -/- BT* mice, and completely absent from wild-type animals.

### BAC transgenesis restores normal catecholamine levels to *Dbh -/-* mice

**Fig 2:**
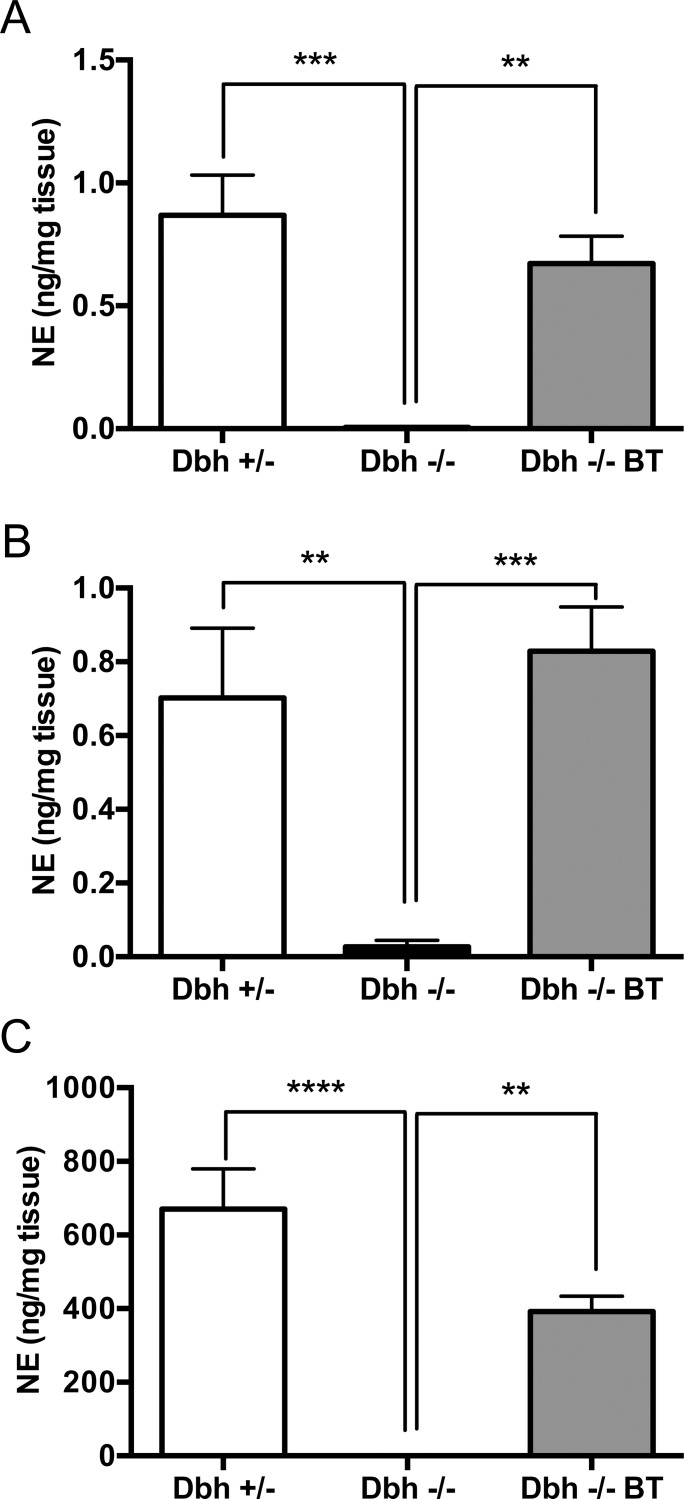
A human BAC transgene restores normal central and peripheral NE levels to *Dbh -/-* mice. *Dbh +/-*, *Dbh -/-*, and *Dbh -/- BT* littermates were assessed for tissue NE levels in the (A) brain, (B) heart, and (C) adrenal by HPLC. Shown is mean ± SEM ng of NE per mg tissue. N = 7–9 per genotype.

**Fig 3:**
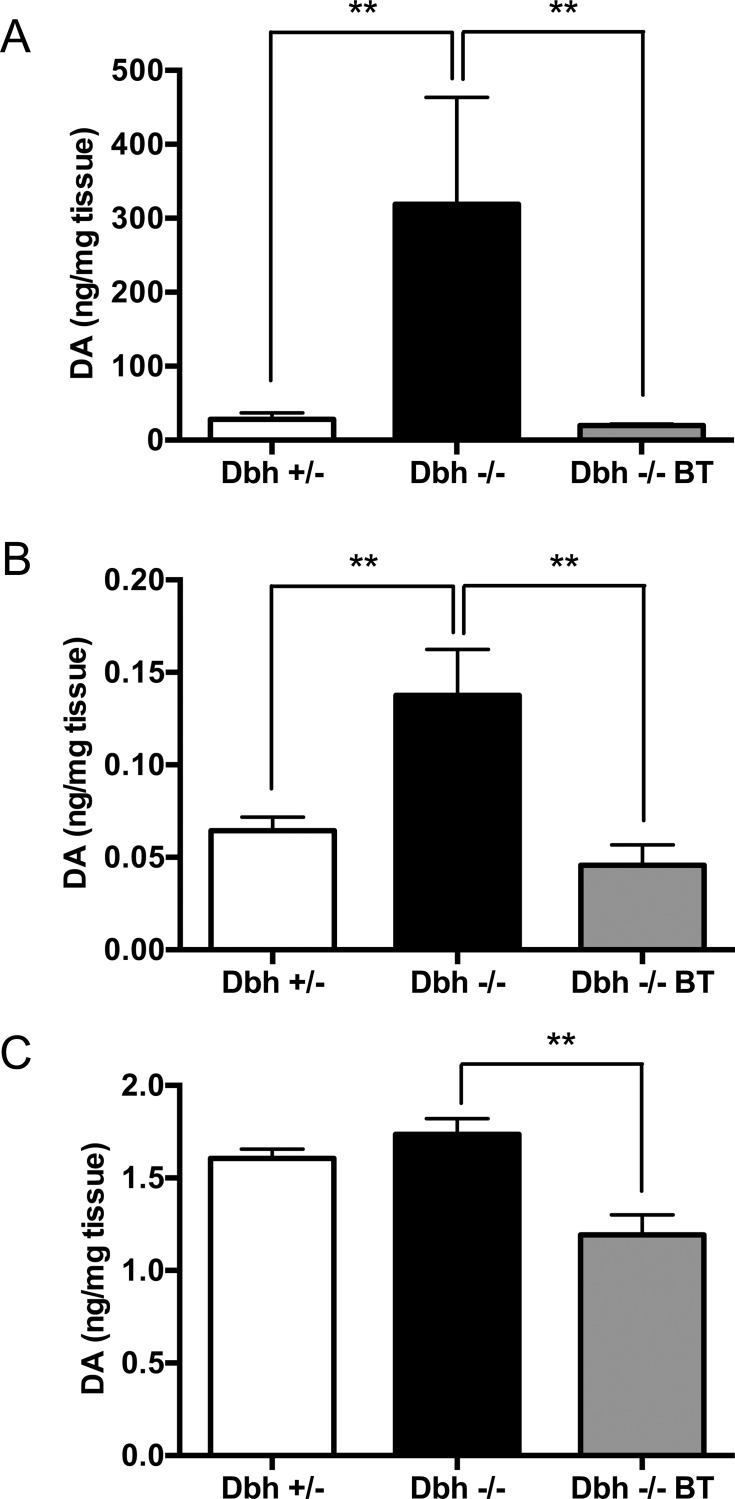
A human BAC transgene restores normal central and peripheral DA levels to *Dbh -/-* mice. *Dbh +/-*, *Dbh -/-*, and *Dbh -/- BT* littermates were assessed for tissue DA levels in the (A) brain, (B) heart, and (C) adrenal by HPLC. Shown is mean ± SEM ng of DA per mg tissue. N = 4–9 per genotype.

DBH is required for the conversion of DA to NE in noradrenergic and adrenergic cells. To determine whether BAC-driven expression of human DBH mRNA resulted in DBH function, NE and DA levels were measured in brain and peripheral organs of DBH-competent control (*Dbh +/-* mice), DBH-deficient (*Dbh -/-*), and transgenic (*Dbh -/- BT*) mice. As described before by us and others [17, 36, 39], *Dbh -/-* mice lack NE and have elevated DA levels in all tissues examined, while the presence of the BAC transgene increased NE and decreased DA close to control levels (Fig 2, Fig 3). One-way ANOVA showed a significant effect of mouse genotype (referring here to *Dbh* genotype with or without BAC transgenesis) for brain (NE: F_2,21_ = 13.20, p<0.001; DA: F_2,19_ = 9.95, p<0.001), adrenal (NE: F_2,21_ = 22.38, p<0.0001; DA: F_2,19_ = 7.57, p<0.01), and heart (NE: F_2,19_ = 13.20, p<0.001; DA: F_2,13_ = 11.04, p<0.01). Tukey’s posthoc tests revealed that NE was significantly reduced and DA was significantly elevated in *Dbh -/-* mice compared to *Dbh +/-* controls, while *Dbh -/- BT* mice had catecholamine content that was significantly different than *Dbh -/-* mice and comparable to controls.

### BAC transgenesis rescues embryonic lethality, growth delay, ptosis, novelty-induced locomotor activity, and seizure susceptibility associated with DBH deficiency

We next determined whether the restoration of normal catecholamine levels by BAC transgenic expression of human DBH rescues physiological and behavioral phenotypes associated with DBH deficiency. Approximately 95% of *Dbh -/-* mice die during embryogenesis or during the first few days of life unless adrenergic receptor agonists and DOPS are added to the drinking water of the pregnant dam [4]. Table 1 shows the numbers of viable offspring born to *Dbh -/- BT* males crossed with *Dbh +/-* dams and untreated with DOPS. The proportion of *Dbh -/-* offspring lacking the transgene was far below Mendelian expectations (χ^2^, 3 d.f. = 36.7, p <0.0001), whereas the BAC construct fully rescued viability in *Dbh -/- BT* offspring, with the proportions of the three viable genotypes not differing from one-third for each genotype (χ^2^, 2 d.f. = 2.21, p = 0.331). By contrast, crosses between *Dbh -/-* male and *Dbh +/-* females that received adrenergic agonists + DOPS during pregnancy resulted in 71 *Dbh +/-* and 43 *Dbh -/-* viable progeny (38%), as compared to 32 *Dbh +/- BT* and 38 *Dbh -/- BT* viable offspring (54%; expected proportions 50% for each set of genotypes; p = 0.03, Fisher’s Exact Test) in the crosses of *Dbh -/- BT* males and untreated *Dbh +/-* females.

**Table 1:** Viable offspring born to a *Dbh +/-* female by *DBH -/-BT* male cross*.

Offspring genotype	Observed Count	Observed proportion	Expected Proportion+
*DBH +/-*	45	0.385	0.25
*DBH +/-BT*	32	0.274	0.25
*DBH -/-*	2	0.017	0.25
*DBH -/-BT*	38	0.325	0.25

*χ2, 3 d.f. = 36.7, p <0.0001.

**+**Mendelian expectation assuming no impact of genotype on survival.

**Fig 4:**
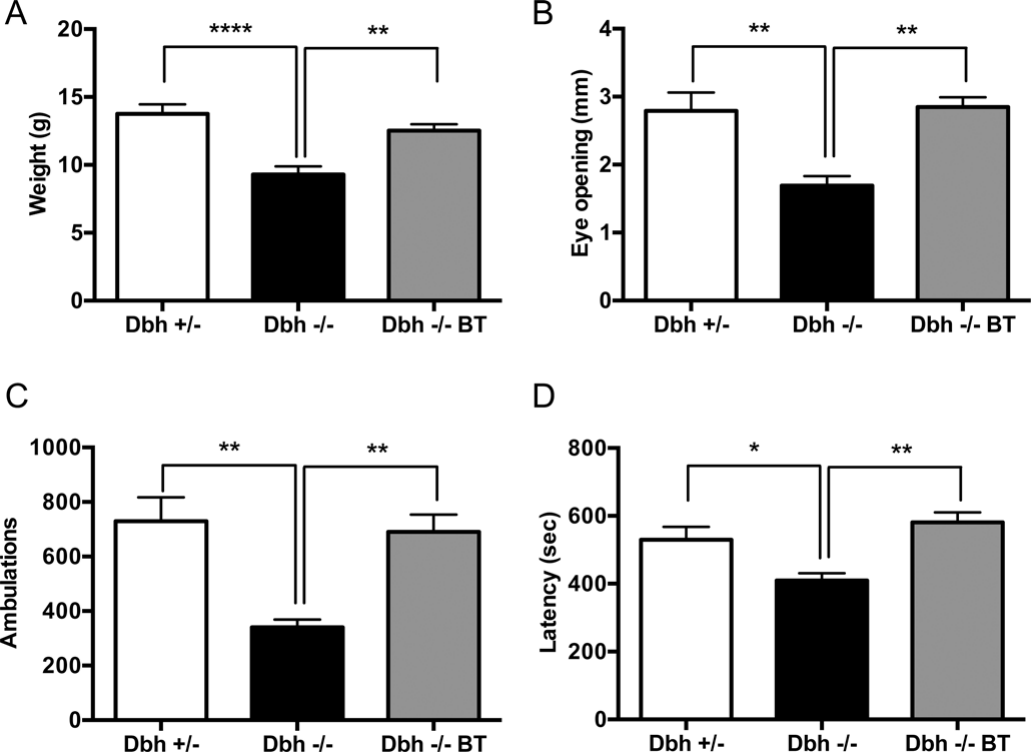
A human BAC transgene rescues *Dbh -/-* developmental, physiological, and behavioral phenotypes. *Dbh +/-*, *Dbh -/-*, and *Dbh -/- BT* littermates were assessed for (A) weaning weight, (B) ptosis, (C) novelty-induced locomotor activity, and (D) seizure susceptibility. Shown is mean ± SEM (A) weight in grams, (B) mm eye opening, (C) ambulations in 30 min, and (D) latency to flurothyl-induced generalized seizure. N = 6–8 per genotype. *p<0.05, **p<0.01, ****p<0.0001.

Fig 4 shows comparisons of weaning weight (4A), the degree of ptosis (4B), novelty-induced locomotion (4C) and latency to seizure after exposure to flurothyl (4D) in *Dbh -/- BT* mice, *Dbh -/-* mice born to DOPS-treated dams from which DOPS was withheld after birth, or NE-competent *Dbh +/-* littermates (which prior studies have shown do not differ from wild-type, *Dbh +/+* mice; [4, 8, 40]). One-way ANOVAs showed significant effects of genotype for weaning weight (F_2,25_ = 14.68, p<0.0001), ptosis (F_2,21_ = 11.07, p<0.001), novelty-induced locomotion (F_2,17_ = 11.13, p<0.001), and seizure susceptibility (F_2,19_ = 6.80, p<0.01). Tukey’s posthoc tests revealed that *Dbh -/-* mice weighed less and had reduced eye opening, exploratory activity, and latency to generalized seizure, and BAC transgenesis fully rescued each of the *Dbh -/-* phenotypes.

### The -970C>T (rs1611115) human polymorphism does not affect BAC transgenic human DBH expression in the mouse

**Fig 5:**
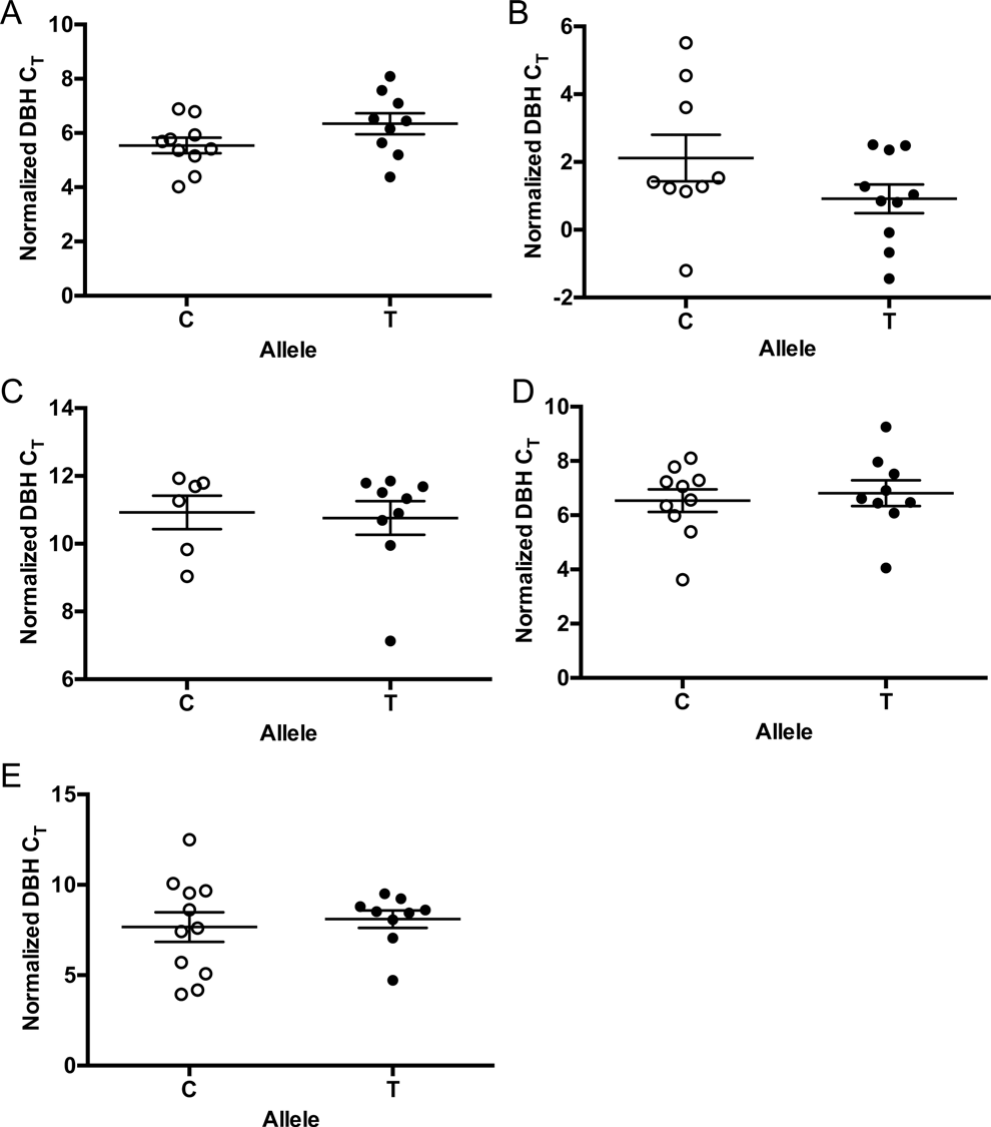
The -970 C-T polymorphism does not affect *Dbh* mRNA abundance. *Dbh -/- BT* mice carrying either the C allele (2 independent lines, pooled) or the T allele (2 independent lines, pooled) at position -970 were assessed for mRNA abundance in the (A) brain, (B) adrenal, (C) heart, (D) liver, and (E) lung by qRT-PCR. Shown are individual and mean ± SEM threshold cycle numbers (ΔCt) normalized to copy number. N = 6–11 per allele.

To determine the specific impact of the -970C>T (rs1611115) human polymorphism on human *DBH* gene expression, we compared mRNA expression in 4 independent *Dbh -/- BT* mouse lines differing only at that single base (2 “C” lines, 2 “T” lines). After controlling for transgene copy number, no significant genotype differences in human *DBH* mRNA abundance were detected for the LC, adrenal, heart, liver, or lung (Fig 5).

## Discussion

DBH catalyzes conversion of DA to NE within noradrenergic and adrenergic vesicles in the central and peripheral nervous systems and in the adrenal medulla. Absence of DBH leads to a syndrome characterized by inadequate sympathetic tone, leading to severe orthostatic hypotension, ptosis and other manifestations of sympathetic failure. Central nervous system manifestations are more subtle in humans, as there are no dramatic psychiatric or neurological manifestations reported in patients with DBH deficiency. However, these patients are chronically medicated with NE-promoting drugs such as DOPS to control the cardiovascular phenotypes, which may also prevent the manifestation of behavioral changes. Although small studies have suggested a variety of links between differences in DBH genotype and/or plasma levels and other human disorders, (reviewed in [41]) more recent genome-wide association studies have not provided evidence for replicable associations of DBH genotype to human neurological or psychiatric disorders (e.g., [42], [43], [44]).

*Dbh* knockout mice have been used to assess brain disorders potentially associated with DBH deficiency. Mice in which targeted disruption of the *Dbh* gene results in absence of DBH and NE display a cardiovascular syndrome highly similar to the human DBH deficiency syndrome [9, 30, 45]. Moreover, several phenotypes with relevance to neurological and neuropsychiatric disorders, including increased seizure susceptibility [8], age-related motor impairment [46], learning and memory deficits [11, 12], decreased arousal/exploration [5, 11], altered antidepressant drug responses [10], and changes in cocaine-induced behaviors [18] occur in *Dbh -/-* mice. The current study demonstrates that introduction of the human *DBH* gene by BAC transgenesis restores the deficits in noradrenergic function resulting from absence of *Dbh* expression, as manifested in organismic, behavioral and neurochemical phenotypes.

The BAC construct drove anatomically specific expression within noradrenergic neurons and the adrenal medulla, demonstrating that the human sequence context surrounding *DBH* was capable of directing the murine transcriptional machinery in a cell-specific manner. That observation was not guaranteed for two reasons. First, while conventional transgenes using small fragments of the human *DBH* promoter are able to drive gene expression in noradrenergic and adrenergic cells, these constructs also result in widespread ectopic expression in non-catecholaminergic neurons and organs [47–49]. Second, the degree of sequence identity between non-coding regions of *DBH* and *Dbh* are relatively modest. For example, comparison of the proximal 2000 bp immediately 5’ to the translational start site (ATG) of human *DBH* (GRC 38/hg 38) and mouse *Dbh* (GRCm38/mm10) using ALIGN [50] (http://atlas.igh.cnrs.fr/bin/align-guess.org) reveals only 52.8% sequence identity.

In contrast to conventional *Dbh -/-* mice, which lack NE completely and have elevated DA levels due to DA production in noradrenergic/adrenergic cells, *Dbh -/- BT* mice had NE and DA levels comparable to controls. In general, transgenic rescue of catecholamine abundance was superior to that achieved via DOPS administration. A thorough characterization of DOPS-induced neurochemical changes in *Dbh -/-* mice was reported by Thomas and colleagues [30]. They showed that a single injection of DOPS (1 mg/g, s.c.) is sufficient to transiently restore NE to wild-type levels in most peripheral organs. However, NE rescue was only partial in most areas of brain and completely deficient in adrenal. Repeated DOPS treatment (1 mg/g, s.c. every 12 hours, 7 injections total) provides extra benefit in some brain regions (e.g. frontal cortex) but not others (e.g. midbrain, cerebellum). Moreover, because DOPS bypasses the requirement for DBH by relying on a different enzyme for NE synthesis (aromatic amino acid decarboxylase; AADC) rather than correcting its deficiency, pharmacological rescue does not attenuate the excessive production of DA in noradrenergic/adrenergic cells. By contrast, the BAC transgene almost completely reversed both the NE deficiency and DA surplus in all tissues examined, including the adrenal gland. Another important difference is that because AADC is expressed in dopaminergic and serotonergic neurons as well as noradrenergic/adrenergic cells, NE production and release following DOPS administration lacks anatomical specificity. The BAC transgene drives DBH expression, and thus NE production and release, in a pattern indistinguishable from the endogenous condition.

Most aspects of physiology and behavior in *Dbh -/-* mice were rescued comparably by either DOPS or transgenesis; for example, both approaches normalized ptosis and seizure susceptibility [8, 30], while the impact of DOPS on novelty-induced locomotor activity and postnatal growth in *Dbh -/-* mice has not been examined. These results suggest that partial DBH or NE deficiency does not have a demonstrative impact on these phenotypes, an idea supported by the lack of any deficit observed in *Dbh +/-* mice that have half the normal copies of *Dbh* but close to normal catecholamine levels. The one exception was pup survival, where the transgene was far superior. Notably, in that case DOPS was administered at a low dose via drinking water to pregnant dams, a paradigm that restores NE levels to only ~10% of normal [4].

Contrary to our original hypothesis, DBH expression in mice carrying the “T” allele at position -970 did not differ appreciably from that in “C” mice. This observation is in line with the report of Barrie and colleagues [28] that expression of mRNA encoding DBH differed only in the liver of humans, but not in the brain, strongly suggesting that -970C>T only impacts human expression of the *DBH* gene within sympathetic noradrenergic neurons [28], and is consistent with the finding that the majority of the serum enzyme arises from the sympathetic nervous system [19]. However, even liver DBH expression did not significantly differ between carriers of the “T” and “C” alleles in *Dbh -/- BT* mice. These results suggest that the transcriptional machinery sensitive to the -970C>T region of the human promoter is not present in mouse noradrenergic sympathetic neurons, or if it is present, the transcriptional machinery in mouse does not respond to the sequence surrounding rs1611115 in the same way the human machinery does. Consistent with that idea is that the sequence immediately surrounding rs1611115 (AGTCTACTTG**[C/T]**GGGAGAGGAC) reveals only modest similarity to the corresponding sequence in mouse (GTTCTCATCA**T**GAGACAGACA, where underlined bases are identical to human sequence and bold shows the base corresponding to rs1611115). Alternatively, despite all of the human association evidence to the contrary [22, 25, 26, 28], it is possible that -970C>T is not the functional polymorphism accounting for the substantial difference in serum DBH levels associated with genotype at this SNP, or that it requires cooperation with distal enhancer sites not present in the transgene. Despite our failure to detect a gene expression difference, this is, to our knowledge, the first use of BAC transgenesis in mice to elucidate the potential function of a neuronal non-coding human SNP, an approach that should prove useful for other variants identified in association studies.
